# General Anesthetics Inhibit LPS-Induced IL-1*β* Expression in Glial Cells

**DOI:** 10.1371/journal.pone.0082930

**Published:** 2013-12-11

**Authors:** Tomoharu Tanaka, Shinichi Kai, Tomonori Matsuyama, Takehiko Adachi, Kazuhiko Fukuda, Kiichi Hirota

**Affiliations:** 1 Department of Anesthesia, Kyoto University Hospital, Kyoto, Japan; 2 Department of Anesthesia, Tazuke Kofukai Medical Research Institute, Kitano Hospital, Osaka, Japan; 3 Department of Anesthesiology, Kansai Medical University, Hirakata, Japan; Université Libre de Bruxelles, Belgium

## Abstract

**Background:**

Glial cells, including microglia and astrocytes, are considered the primary source of proinflammatory cytokines in the brain. Immune insults stimulate glial cells to secrete proinflammatory cytokines that modulate the acute systemic response, which includes fever, behavioral changes, and hypothalamic-pituitary-adrenal (HPA) axis activation. We investigated the effect of general anesthetics on proinflammatory cytokine expression in the primary cultured glial cells, the microglial cell line BV-2, the astrocytic cell line A-1 and mouse brain.

**Methodology/Principal Findings:**

Primary cultured glial cells were exposed to lipopolysaccharide (LPS) in combination with general anesthetics including isoflurane, pentobarbital, midazolam, ketamine, and propofol. Following this treatment, we examined glial cell expression of the proinflammatory cytokines interleukin (IL)-1*β*, IL-6, and tumor necrosis factor-alpha (TNF-*α*). LPS-induced expression of IL-1*β* mRNA and protein were significantly reduced by all the anesthetics tested, whereas IL-6 and TNF-*α* mRNA expression was unaffected. The anesthetics suppressed LPS-induced extracellular signal-regulated kinase 1/2 (ERK 1/2) phosphorylation, but did not affect nuclear factor-kappaB and activator protein-1 activation. The same effect was observed with BV-2, but not with A-1 cells. In the mouse experiments, LPS was injected intraperitoneally, and isoflurane suppressed IL-1*β* in the brain and adrenocorticotropic hormone in plasma, but not IL-1*β* in plasma.

**Conclusions/Significance:**

Taken together, our results indicate that general anesthetics inhibit LPS-induced IL-1*β* upregulation in glial cells, particularly microglia, and affects HPA axis participation in the stress response.

## Introduction

Humans and animals respond rapidly to infection by activating their innate immune system. Immune-related messages relayed from the periphery to the brain activate neural pathways that regulate the acute phase response, which includes fever, behavioral depression, and hypothalamic-pituitary-adrenal (HPA) axis activation [1]. These responses, which are organized to fight against the infection, are triggered by proinflammatory cytokines such as interleukin (IL)-1*β*, tumor necrosis factor-alpha (TNF-*α*), and IL-6. These cytokines are secreted by activated neutrophils and monocytes that contact the invading microorganisms [2]. Peripheral immune signals are communicated to the brain by neural or humoral routes [3]. The neural route is mediated by the vagus nerve, which includes sensory neurons that express IL-1 receptors [4]. The humoral route stimulates brain regions that lack the blood–brain barrier and react to cytokines or pathogen-associated molecular patterns [3]. These responses begin in the circumventricular organs or choroid plexus and then diffuse to other brain regions [3]. Information transmitted over either pathway causes the brain to produce same kind of proinflammatory cytokines produced in periphery [5]. Among these cytokines, IL-1*β* is considered the primary regulator of the systemic response to infection. Central administration of IL-1*β* induces all components of the acute phase reaction, including fever, HPA axis activation, and behavioral depression [6], whereas IL-6 has no behavioral activity [7].

Glial cells, including microglia and astrocytes, are the primary source for proinflammatory cytokines in the brain. Microglia are resident macrophage-like cell population and are considered to play a pivotal role in the brain’s innate immune response [8]. Under normal conditions, microglia are quiescent and scattered [9]. Occasionally, microglia are moderately activated as scavengers to maintain and restore the brain [10]. In the case of systemic infection, microglia are activated and release proinflammatory cytokines to initiate acute inflammatory responses. Minocycline, a microglial inhibitor, attenuates lipopolysaccharide (LPS)-induced sickness behaviors [11]. According to a recent report, astrocytes can release proinflammatory molecules and modulate immune responses [12]. Therefore, microglia and astrocytes are considered major components that mediate immune responses and inflammation in the brain [13].

In clinical settings, general anesthetics are typically administered to infectious patients for surgical procedures, but also for sedation with critical care. Ketamine and dexmedetomidine can inhibit LPS-induced microglial activation; however, few studies have examined whether general anesthetics affect the ability of glial cells to produce proinflammatory cytokines [9,14]. In addition, the effect of anesthetics on astrocyte cytokine production is poorly understood. Recently, we reported that various general anesthetics also inhibit glial cell production of erythropoietin under hypoxic conditions, which suggests that general anesthetics have a common direct effect on glial cell functions [15]. Therefore, in the present study, we investigated the effects of several general anesthetics, including isoflurane, pentobarbital, midazolam, ketamine, and propofol, on LPS-induced upregulation of proinflammatory cytokines in primary cultured glial cells. Considering the pivotal role that proinflammatory cytokines play during the brain’s acute inflammation phase, the influence of general anesthetics on cytokine production from glial cells may change the systemic response to infection involving HPA axis. Therefore, we performed an additional experiment *in vivo* in which we intraperitoneally injected mice with LPS. We then evaluated cytokine induction in the brain and adrenocorticotropic hormone (ACTH) concentration in plasma to determine whether general anesthetics affected the systemic response to infection.

## Results

### Anesthetics suppress LPS-induced upregulation of IL-1*β* mRNA and protein in primary cultured glial cells

**Figure 1:**
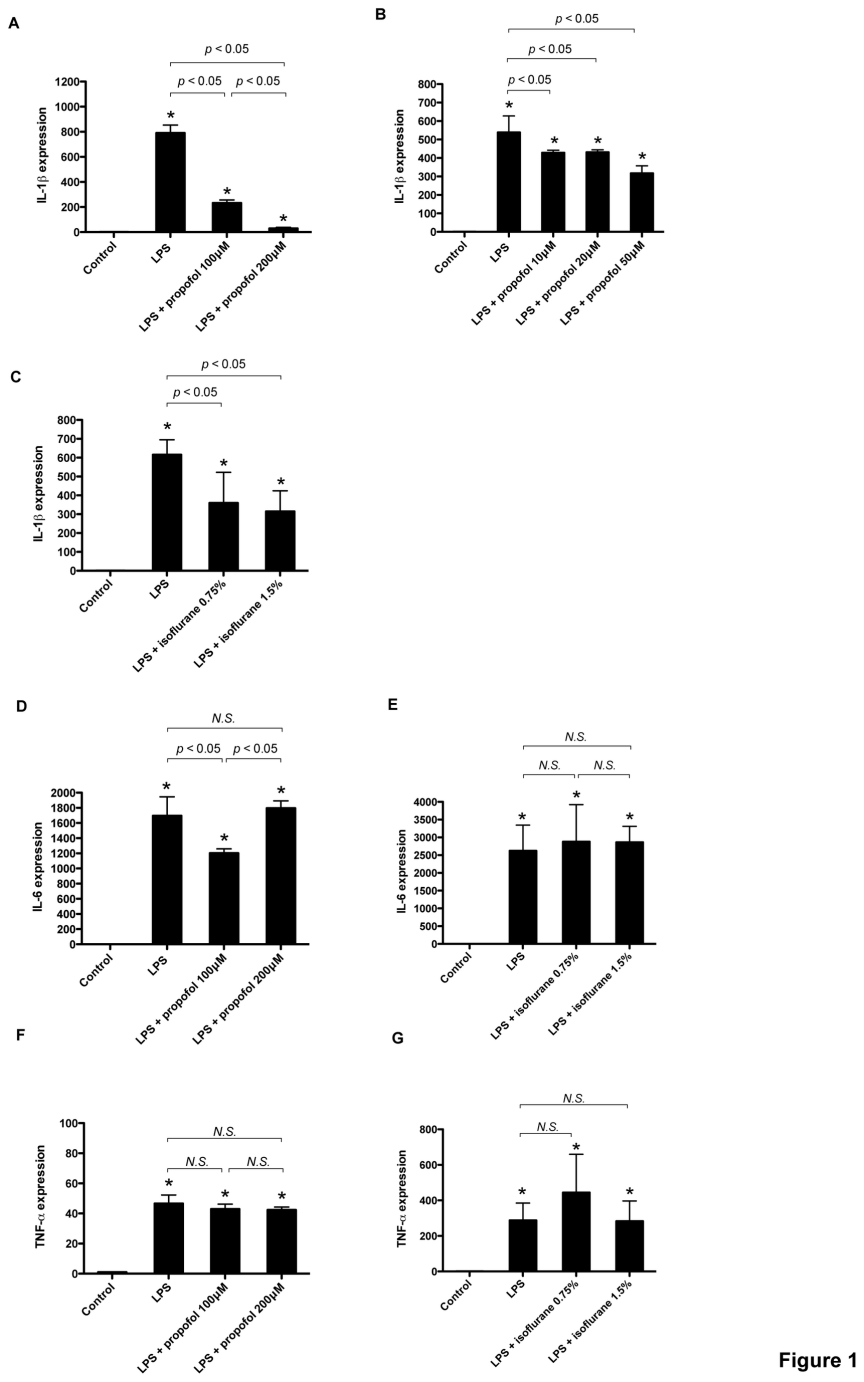
Effects of propofol and isoflurane on proinflammatory cytokine expression in cultured glial cells. Primary cultured glial cells were exposed for 4 hours to lipopolysaccharide (LPS) (1 µg/ml) and propofol or isoflurane at the indicated concentrations. Interleukin (IL)-1*β* (A, B, C), IL-6 (D, E), and tumor necrosis factor-alpha (TNF-α) (F, G) mRNA were assayed with real-time RT-PCR. Data are presented as mean ± SD (n = 4). The expression levels of IL-1*β*, IL-6, and TNF-*α* were normalized to that of 18S, and were expressed relative to the control mean. *P < 0.05 versus control, N.S., not significant (Mann–Whitney U-test).

**Figure 2:**
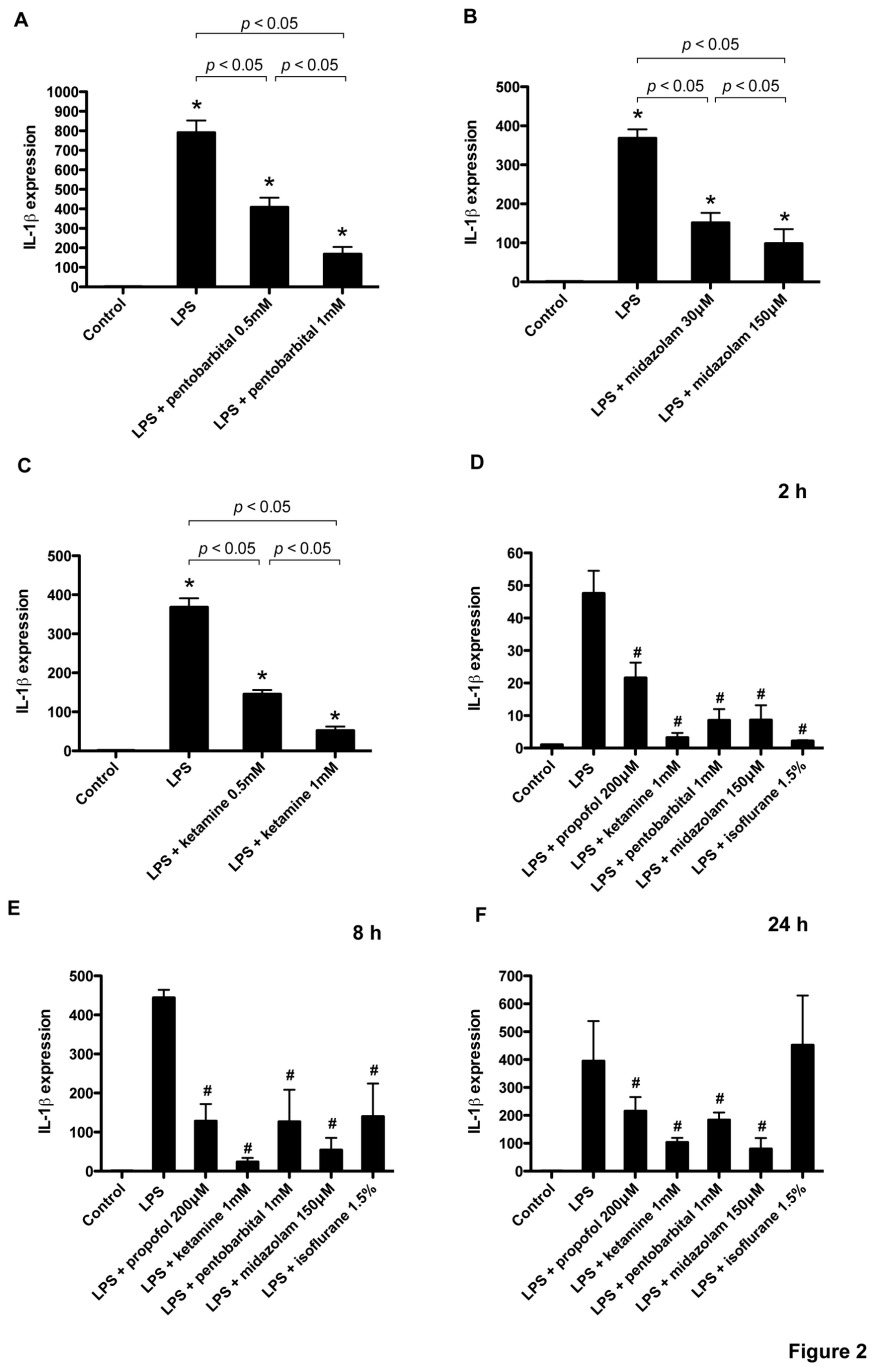
Effects of anesthetics on LPS-induced IL-1*β* upregulation in cultured glial cells. Primary cultured glial cells were exposed for 4 hours to LPS (1 µg/ml) in the presence of pentobarbital (A), midazolam (B), or ketamine (C) at the indicated concentrations, or in the presence of propofol (200 µM), ketamine (1 mM), pentobarbital (1 mM), midazolam (150 µM), or isoflurane (1.5%) for 2 (D), 8 (E), or 24 (F) hours. IL-1*β* mRNA was assayed with real-time RT-PCR. Data are presented as mean ± SD (n = 6). The expression levels of IL-1*β* were normalized to that of 18S and expressed relative to the control mean. *P < 0.05 versus control, # *P* < 0.05 versus LPS, *N.S.*, not significant (Mann–Whitney U-test).

**Figure 3:**
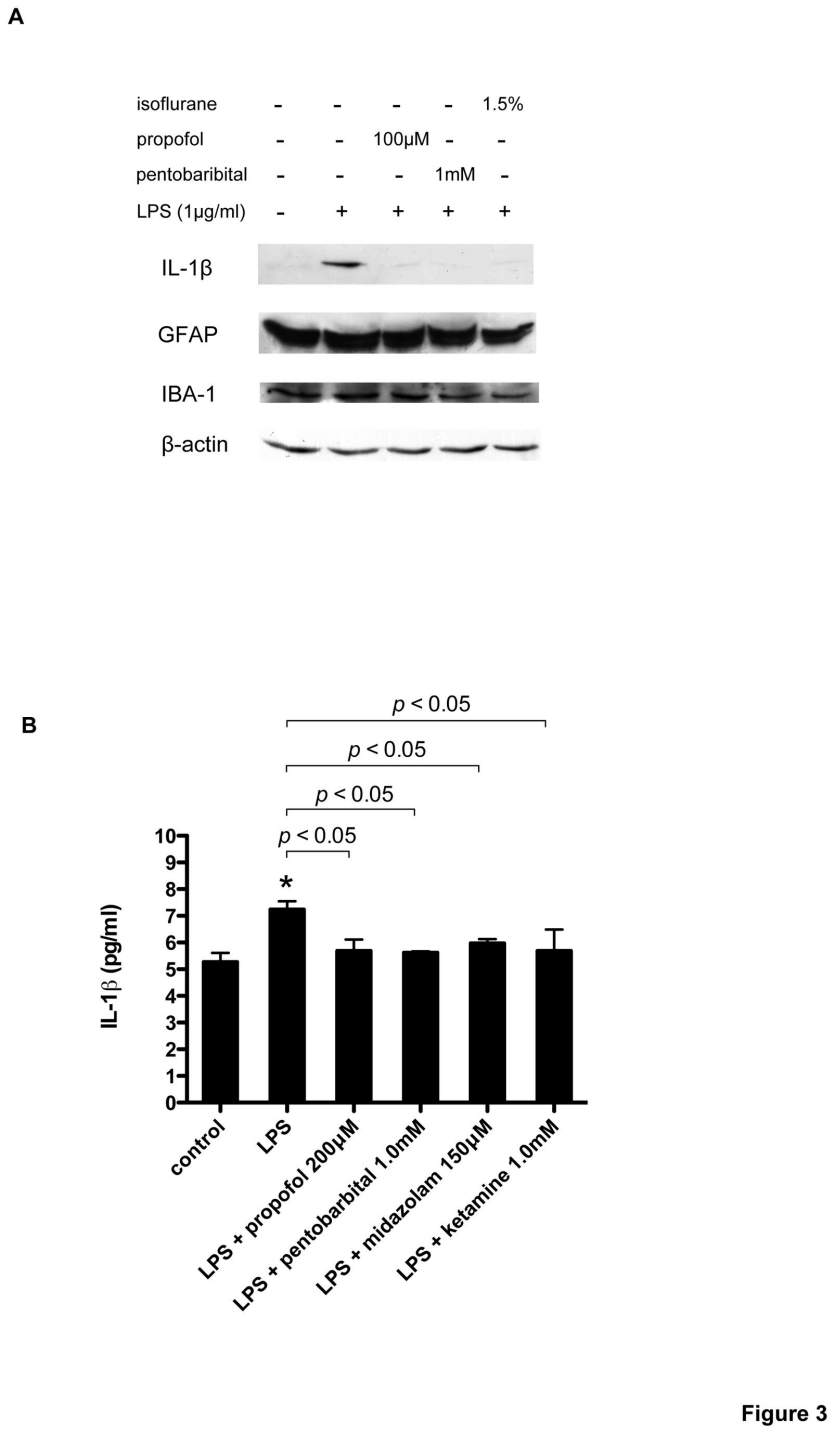
Effects of anesthetics on LPS-induced IL-1*β* protein expression and secretion in cultured glial cells. Primary cultured glial cells were exposed for 4 hours to LPS (1 µg/ml) with or without 1.5% isoflurane, 100 µM propofol, or 1 mM pentobarbital. (A) Whole cell lysates were analyzed for IL-1*β*, glial fibrillary acidic protein (GFAP), ionized calcium-binding adapter molecule 1 (IBA-1), and *β*-actin protein expression by immunoblot assay. Figures are representative of at least three independent experiments. (B) IL-1*β* protein concentration in cultured medium was measured with ELISA. Data are presented as mean ± SD (n = 3). *P < 0.05 versus control (Mann–Whitney U-test).

Primary cultured glial cells were exposed to LPS (1 µg/ml) with propofol or isoflurane for 4 h. LPS exposure significantly induced IL-1*β* mRNA upregulation, which was suppressed by propofol and isoflurane (Figure 1A-C). LPS induced IL-6 and TNF-*α* mRNA upregulation, but isoflurane and propofol failed to suppress their induction, except with a propofol concentration of 100 µM that suppressed IL-6 induction (Figure 1D-G). Next, to examine whether the effects of propofol and isoflurane was observed with other general anesthetics, we exposed glial cells to LPS with pentobarbital, midazolam, and ketamine. As with propofol and isoflurane, these anesthetics suppressed LPS-induced IL-1*β* upregulation (Figure 2A–C). To determine whether these effects changed over time, we performed the same experiments at 2, 8, and 24 h. At 2 h and 8 h, all anesthetics suppressed LPS-induced IL-1*β* induction (Figure 2D–F). To investigate the IL-1*β* induction at the protein level, we analyzed IL-1*β* protein accumulation in whole cell lysates obtained from glial cells. An immunoblot assay showed remarkable induction of IL-1*β* protein with LPS, and its expression was significantly suppressed with isoflurane, propofol, and pentobarbital (Figure 3A). Finally, IL-1*β* protein secretion in medium was assayed with enzyme-linked immunosorbent assay (ELISA). As shown in Figure 3B, IL-1*β* protein concentration in cultured medium was significantly elevated after a 4-h LPS exposure (1 µg/ml), and the anesthetics propofol, pentobarbital, midazolam, and ketamine suppressed this elevation.

### Propofol, isoflurane, and pentobarbital inhibit LPS-induced phosphorylation of ERK, but do not affect NF-κB or AP-1 activation

**Figure 4:**
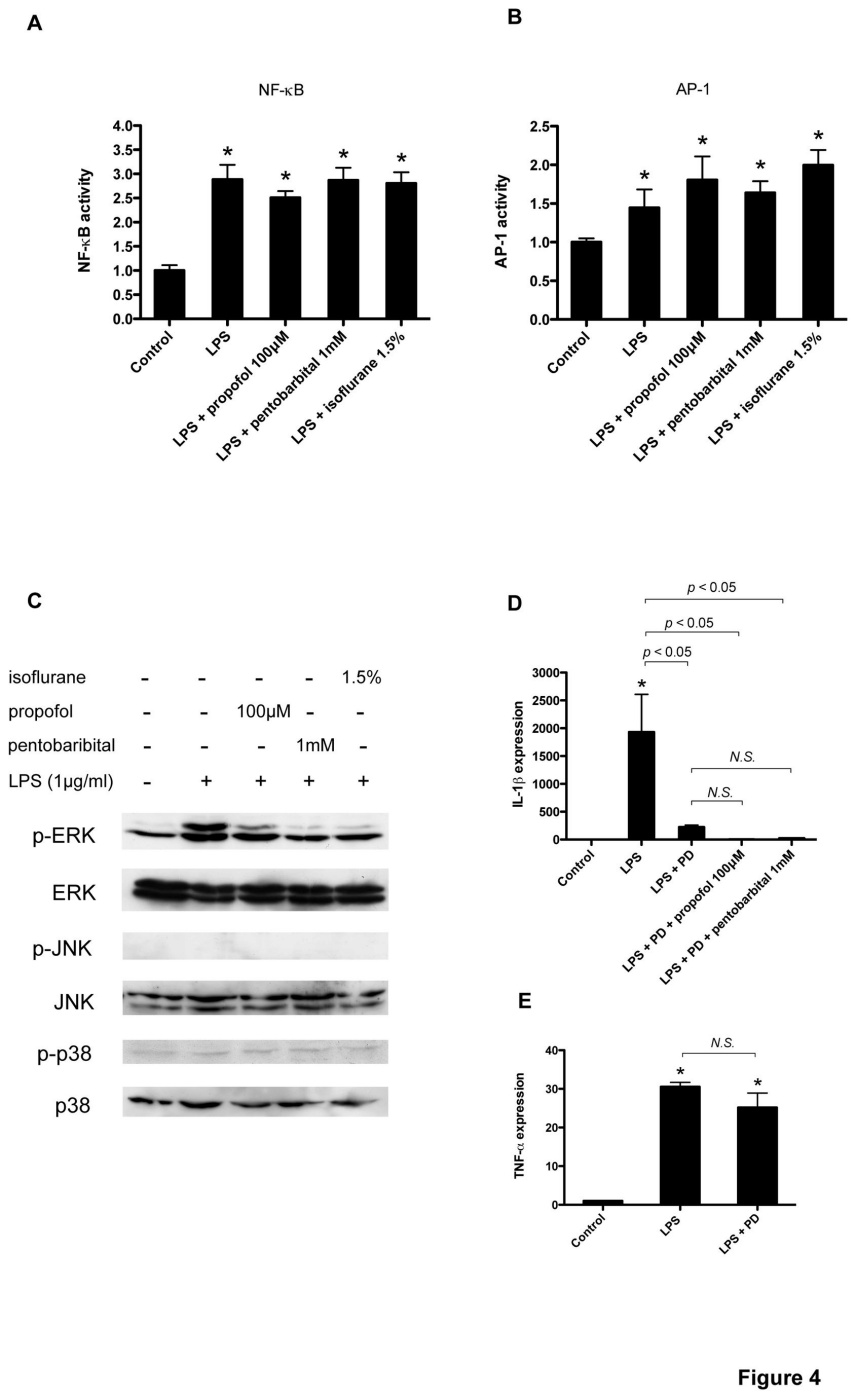
Mechanism underlying inhibitory effect of anesthetics on IL-1*β* upregulation in LPS-treated cultured glial cells. (A, B) Effect of anesthetics on nuclear factor-kappaB (NF-κB) and activator protein-1 (AP-1) transcription in LPS-treated cultured glial cells. Primary cultured glial cells were exposed to LPS (1 µg/ml) with or without 100 µM propofol, 1 mM pentobarbital, or 1.5% isoflurane for 4 h. NF-κB (A) and AP-1 (B) transcriptional activities were measured with an ELISA-based kit. Data are presented as mean ± SD (n = 3). *P < 0.05 versus control. No statistically significant difference was found between any two groups except with the control. (Mann–Whitney U-test) (C) Effect of anesthetics on activity of MAPK families in LPS-treated cultured glial cells. Primary cultured glial cells were exposed to LPS (1 µg/ml) with or without 1.5% isoflurane, 100 µM propofol, or 1 mM pentobarbital for 4 hours. Whole cell lysates were analyzed for extracellular signal-regulated kinase (ERK), phospho-ERK (p-ERK), phospho-JNK (p-JNK), JNK, phospho-p38 (p-p38), and p38 MAPK expression by immunoblot assay. Figures are representative of at least three independent experiments. (D, E) Effect of PD98059, an antagonist of ERK 1/2, on IL-1*β* and TNF-*α* expression in LPS-treated cultured glial cells. IL-1*β* and TNF-*α* mRNA was assayed with real-time RT-PCR. Primary cultured glial cells were exposed to LPS (1 µg/ml) and 50 µM PD98059 (PD) with or without 100 µM propofol or 1 mM pentobarbital, and harvested for 4 h. IL-1*β* and TNF-*α* mRNA was assayed with real-time RT-PCR. Data are presented as mean ± SD (n = 3). The expression levels of IL-1*β* and TNF-*α* were normalized to that of 18S and expressed relative to the mean of control. *P < 0.05 versus control, *N.S.*, not significant (Mann–Whitney U-test).

LPS activates nuclear factor-kappaB (NF-κB), activator protein-1 (AP-1), and mitogen-activated protein kinases (MAPKs), which have been implicated in proinflammatory cytokine release [16,17]. To investigate the mechanism through which anesthetics suppress LPS-induced IL-1*β* upregulation, we examined the influence of anesthetics on NF-κB and AP-1 activity in glial cells. NFκB and AP-1 transcription quantified with an ELISA-based kit was significantly elevated with LPS exposure, and propofol, pentobarbital, and isoflurane failed to suppress such activity (Figure 4A, B). Next, we examined MAPK activity in glial cells, which revealed three major MAPK signaling molecules: extracellular signal-regulated kinase 1/2 (ERK 1/2), c-Jun N-terminal protein kinase (JNK), and p38 MAPK. Based on these findings, we performed a western blot analysis using the phospho- or total form antibodies against ERK 1/2, JNK, and p38 MAPK. We observed that propofol, pentobarbital, and isoflurane remarkably decreased LPS-stimulated phosphorylation of ERK1/2 at 4 h, but had no effect on total ERK 1/2 expression (Figure 4C). Anesthetics had no effect on phosphorylation or total expression of JNK or p38 MAPK (Figure 4C). Finally, in order to examine ERK involvement in LPS-induced IL-1*β* upregulation, the ERK inhibitor PD98059 was added to the cultured glial cells. As shown in Figure 4D, PD98059 remarkably suppressed LPS-induced IL-1*β* mRNA upregulation, and the anesthetics pentobarbital and propofol did not exert an additive effect with PD98059. In contrast, PD98059 did not suppress LPS-induced TNF-*α* mRNA upregulation (Figure 4E).

### Propofol and isoflurane suppress LPS-induced upregulation of IL-1*β* mRNA in the BV-2 microglial cell line, but not in the A-1 astrocytic cell line

**Figure 5:**
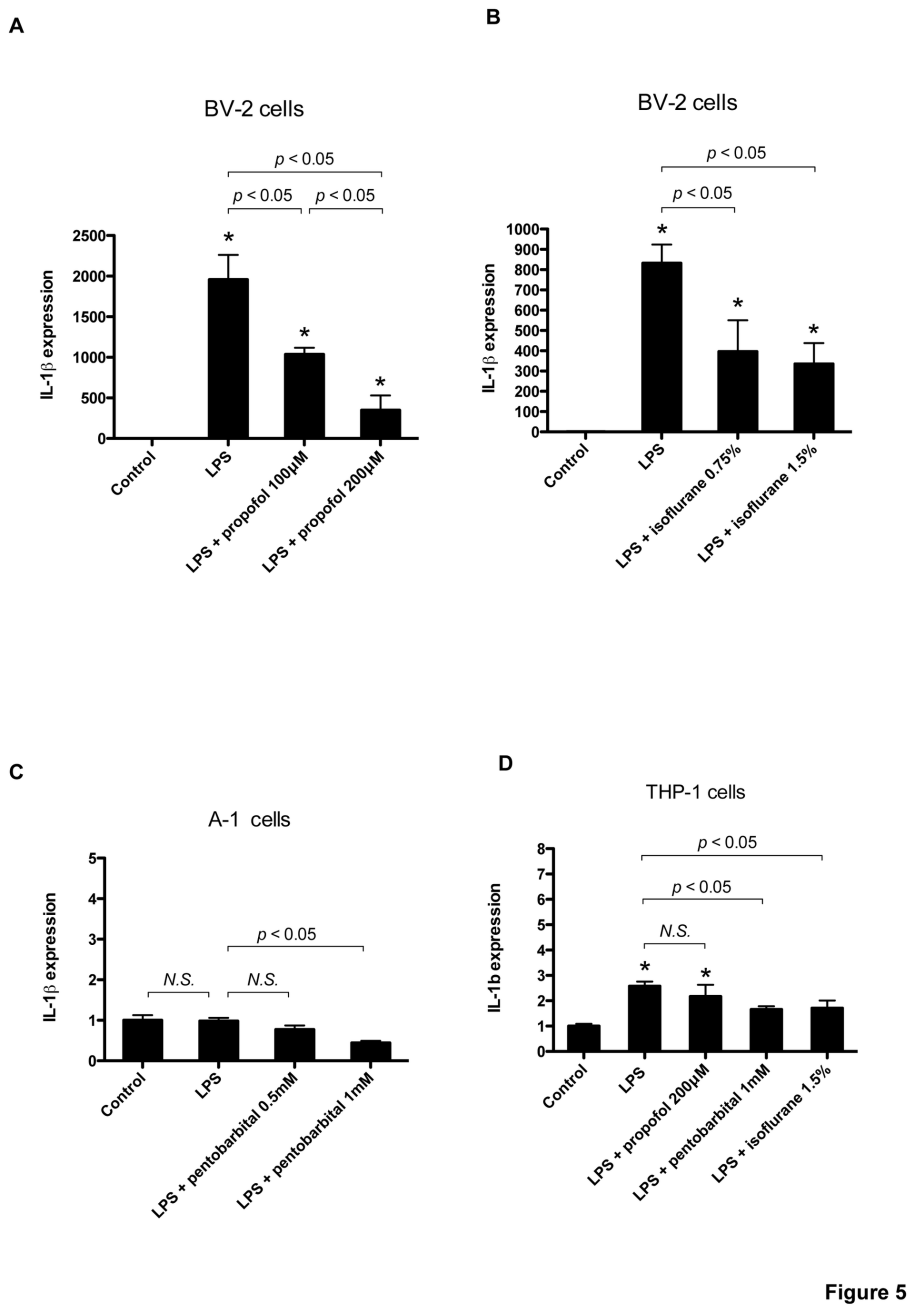
Effect of anesthetics on LPS-induced IL-1*β* upregulation in BV-2, A-1, and THP-1 cells. BV-2 microglial cells were exposed to LPS (1 µg/ml) in the presence of the indicated concentrations of propofol (A) or isoflurane (B) for 4 hours. IL-1*β* mRNA was assayed with real-time RT-PCR. Data are presented as mean ± SD (n = 3). A-1 astrocytic cells, were exposed to LPS (1 µg/ml) in the presence of the indicated concentrations of pentobarbital for 4 hours. Human acute monocytic leukemia cells, or THP-1 cells, were exposed to LPS (1 µg/ml) in the presence of the indicated concentrations of pentobarbital or isoflurane for 4 hours. IL-1*β* expression levels were normalized to that of 18S and expressed relative to the control mean. *P < 0.05 versus control, *N.S.*, not significant (Mann–Whitney U-test).

For this experiment, we used cultures that included astrocytes, oligodendrocytes, neurons, microglia, and ependymal cells, although astrocytes were the predominant cell type [18]. Microglia and astrocytes are considered candidates for the source of proinflammatory cytokines, [19]. As is shown in Figure 3B, we detected in an immunoblot the astroglial marker, glial fibrillary acidic protein (GFAP), and the microglial marker, ionized calcium-binding adapter molecule 1 (IBA-1). However, the neuron marker, neuronal nuclei, was not detected (data not shown). To determine which cells are the primary sources of cytokines and the cells affected by general anesthetics, we performed the same series of experiments in the BV-2 microglial and A-1 astroglial cell lines. In BV-2 cells, LPS exposure significantly induced IL-1*β* mRNA, but propofol and isoflurane suppressed its induction (Figure 5A, B). In contrast, LPS-induced upregulation of IL-1*β* was not confirmed in A-1 cells, although pentobarbital (1 mM) slightly suppressed IL-1*β* mRNA levels (Figure 5C). Finally, to determine the effect of anesthetics on IL-1*β* induction by hematopoietic cells, we used the human monocytic cell line THP-1. Following differentiation with phorbol-12-myristate-13-acetate (PMA), THP-1 cells showed significant induction of IL-1*β* mRNA with LPS, and pentobarbital and isoflurane suppressed this induction, but to a lesser extent than was observed with BV-2 cells (Figure 5D). THP-1 without PMA did not show IL-1*β* mRNA induction with LPS (data not shown). 

### Isoflurane inhibits IL-1*β* expression in the mouse brain after LPS treatment

**Figure 6:**
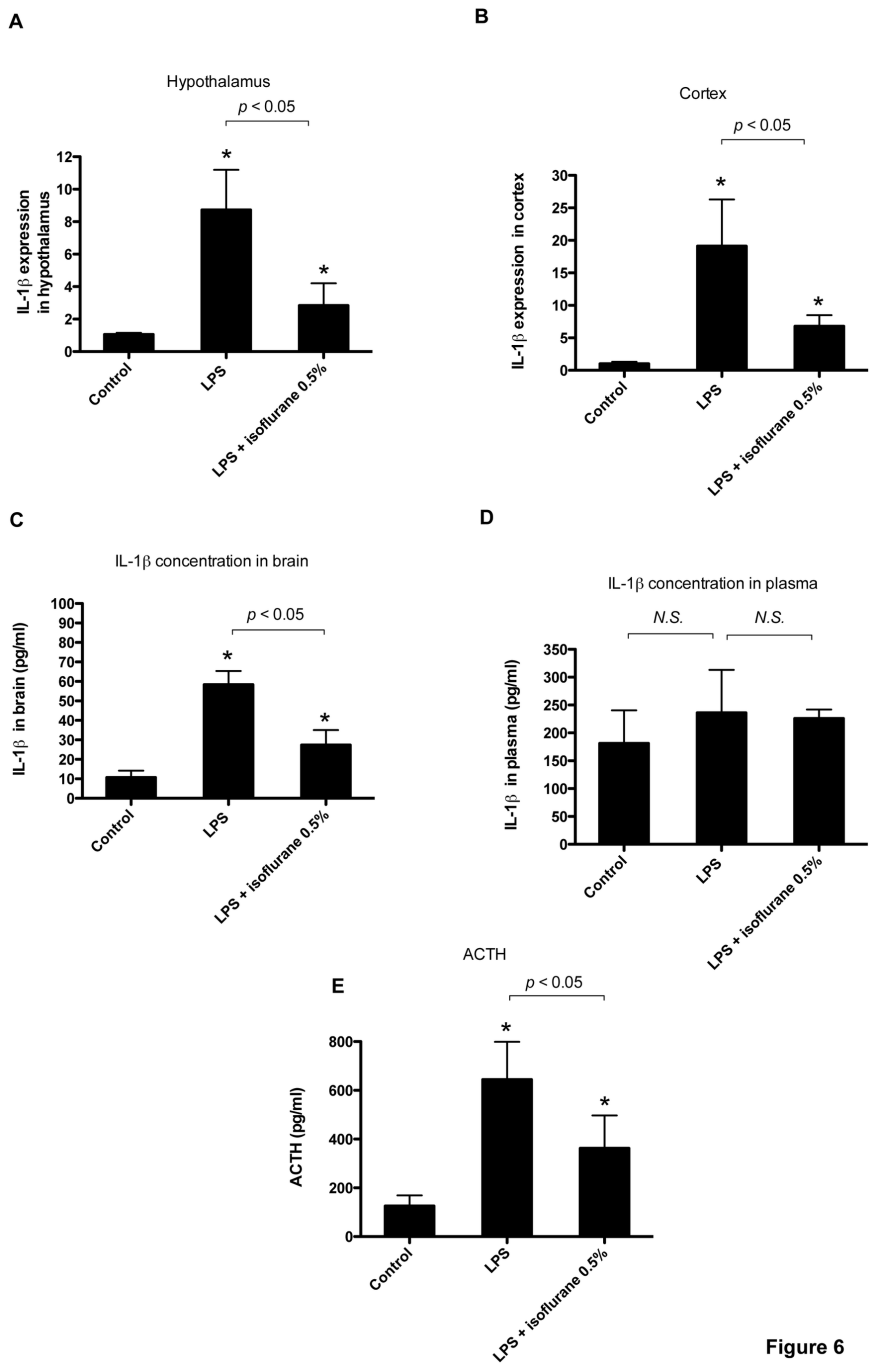
Effect of isoflurane on LPS-induced IL-1*β* expression in mice, and plasma adrenocorticotropic hormone (ACTH) levels. Ten-week-old BALB/c mice were exposed to 0.5% isoflurane for 4 hours after LPS treatment (5 mg/kg) (n = 6). IL-1*β* mRNA in the hypothalamus (A) and cortex (B) was assayed with real-time RT-PCR analysis. IL-1*β* expression levels were normalized to that of 18S and expressed relative to the mean of control mice. IL-1*β* protein concentration (pg/ml) in the brain (C) and plasma (D) was quantified with ELISA. Data are presented as mean ± SD. Serum ACTH concentration (pg/ml) was quantified with ELISA. Data are presented as mean ± SEM. *P < 0.05 versus control; *N.S.*, not significant (Mann–Whitney U-test).

To examine the effect of general anesthetics on IL-1*β* expression in the brain, we intraperitoneally injected LPS (5 mg/kg) in 10-week-old C57BL/6N mice that were subsequently exposed to 0.5% isoflurane for 4 h. LPS treatment significantly increased IL-1*β* mRNA expression, whereas isoflurane suppressed LPS-induced IL-1*β* expression in the hypothalamus (Figure 6A) and cortex (Figure 6B). To confirm the effects of isoflurane at the protein level, we measured IL-1*β* protein concentration in the brains using ELISA. LPS treatment induced a significant increase in IL-1*β* protein in the whole brain 4 h after the LPS treatment, and isoflurane exposure significantly suppressed its induction (Figure 6C). However, IL-1*β* protein concentration in plasma was not elevated at the 4 h time point, and the effect of isoflurane was not apparent (Figure 6D). Because IL-1*β* was linked to HPA axis activation [20], we examined whether anesthetics affected the HPA axis. We measured serum ACTH levels and found a significant increase 4 h after the LPS treatment and that isoflurane exposure significantly suppressed the ACTH elevation (Figure 6E).

## Discussion

Glial cells, including astrocytes and microglia, are the major components that mediate immune responses and inflammation in the brain [21,22]. Glial cells can produce cytokines, reactive oxygen radicals, and nitric oxide in response to infectious insults [23]. In the current study, we demonstrated that anesthetics, including propofol, isoflurane, ketamine, pentobarbital, and midazolam, inhibit the induction of IL-1*β* from glial cells stimulated with LPS. The same effect was confirmed with the BV-2 microglial cell line. Microglia are the main source of inflammatory cytokines in the brain [24]. Therefore, our results suggest that anesthetics exert their IL-1*β* inhibitory effect through a direct effect on microglia. Although several reports indicate anesthetics affect microglia secretion of proinflammatory cytokines [9,14,23,25], the results vary among the types of cytokines or stimulation methods. For example, isoflurane exposure increased the level of the proinflammatory cytokine IL-1*β* in the mice brain [25], whereas ketamine suppressed LPS-induced TNF-*α* production in cultured microglia [23]. We found the anesthetics examined in the present study inhibited IL-1*β* secretion from LPS-stimulated glial cells. Dexmedetomidine [14] and ketamine [9] were reported to have the same effect as isoflurane. Therefore, most clinically used anesthetics have a similar suppressive effect on glial cell induction of IL-1*β*. The primary target of general anesthetics depends on the anesthetic. For example, ketamine acts on N-methyl-D-aspartate receptors [26,27], whereas the volatile anesthetics, propofol and the barbiturates, act on γ-aminobenzoic acid-A receptors [28,29]. On the other hand, the action of general anesthetics on glial cells is poorly understood. Our finding that general anesthetics have an IL-1*β-*suppressive effect *in vitro* indicates that general anesthetics have a common effect on glial cells, particularly microglia. This finding is surprising considering the diverse mechanisms of general anesthetics.

MAPKs have been shown to play an important role in LPS-induced proinflammatory cytokine release [30]. Therefore, we investigated the effect of anesthetics on the activation of three MAPKs and found that the LPS-induced phosphorylation of ERK1/2 in glial cells treated with LPS was suppressed with propofol, isoflurane, and pentobarbital. In contrast, phosphorylation of JNK or p38 MAPK was not affected with LPS or anesthetics. The reason why LPS failed to activate JNK and p38 MAPK may be because the duration of LPS exposure in our experiments (4 h) was too long. In previous reports, activation was observed with less incubation time [31]. In addition, the ERK1/2 inhibitor PD98059 dramatically inhibited LPS-induced IL-1*β* expression, and anesthetics failed to show additive effects. This result indicates that ERK 1/2 activation mainly regulated IL-1*β* induction in LPS-stimulated glial cells in our experiment and that anesthetics typically exert their inhibitory effect by inhibiting ERK 1/2 phosphorylation. On the other hand, the transcription factors NF-κB and AP-1 regulate inflammatory cytokine transcription [32]. Therefore, we investigated the transcriptional activity of NF-κB and AP-1 with an ELISA-based kit in LPS-stimulated glial cells. LPS treatment induced NF-κB and AP-1 activation, but anesthetics did not inhibit their activation. The effect of anesthetics on NF-κB and AP-1 are not well studied, although isoflurane is reported to suppress NF-κB activity in a reperfusion model of the mouse kidney [33].

Recent reports focus on the importance of inflammasome, a multiprotein platform that activates caspase 1, in regulating of IL-1*β* processing and secretion [34–36]. Inflammasome is a multiprotein oligomer that is comprised of a Nod-like receptor (NLR) family, which includes NLRP3, the cytosolic receptor AIM2, and pro-caspase-1 [34]. NLRs activated by bacterial toxins, crystals, and danger associated danger patterns assemble and oligomerize into a common structure that collectively activates the caspase-1 cascade [37]. Once activated, caspase-1 proteolytically cleaves the cytokine precursor pro-IL-1*β* and releases the biologically active form of IL-1*β* [37]. In our experiments, immunoblot analysis using the whole cell lysate of glial cells detected the precursor for IL-1*β*, which has a molecular weight of 32 kd. IL-1*β* in the supernatant of glial cell culture medium, which is considered the mature form, was significantly suppressed by the anesthetics. Therefore, anesthetic inhibition of IL-1*β* was not limited to the transcriptional level, although the effect of anesthetics on inflammasome activity is yet to be determined.

Proinflammatory cytokines produced by glial cells can have both beneficial and harmful effects [31]. Glial cells can produce cytokines, reactive oxygen radicals, and nitric oxide in response to ischemic, traumatic, and infectious insults, and in cases when such inflammatory response is excessive or prolonged, the disease process is exaggerated [31]. Suppressing the inflammatory responses of glial cells attenuates some of these pathological conditions [38,39]. In contrast, proinflammatory cytokines produced by glial cells in the acute phase could be necessary for mammals to respond rapidly to an infection [31]. That is, HPA axis activation and subsequent glucocorticoid production are critical for survival as they regulate the immune system’s responses [40]. Recently, proinflammatory cytokines, particularly IL-1*β* secreted by glial cells, are considered essential for HPA axis response [41]. Therefore, we investigated whether anesthetics affected IL-1*β* expression in the mouse brain 4 h after LPS treatment. We observed IL-1*β* expression in the hypothalamus and cortex was significantly suppressed with isoflurane exposure. Interestingly, serum IL-1*β* concentration was not affected by isoflurane, although brain IL-1*β* concentration was significantly lowered. This finding may be attributed to the difference between microglia and monocytes in their responses to LPS. The LPS-induced IL-1*β* upregulation observed in THP-1 cells was not as distinct as the response observed in BV-2 cells. In contrast, serum ACTH level was increased with LPS treatment, but was suppressed with isoflurane. This result demonstrates that isoflurane affects the HPA axis response. HPA axis contributions to the stress response are essential in the acute phase of infection [40,42,43]. Therefore, our result suggests that anesthetics may have a great impact on the clinical course of septic patients. In fact, septic patients commonly need sedative drugs during critical care. However, according to the recent reports, anesthetics used for such sedation may worsen the prognosis for these critically ill patients [44]. The precise mechanisms underlying the worsened prognosis are largely unknown. Considering the pivotal role the HPA axis plays in the stress response, anesthetics may cause adverse effects by suppressing corticosterone production. Further *in vivo* studies using propofol or midazolam are required to examine this topic further. On the other hand, prolonged glial cell activation and proinflammatory cytokine production can cause chronic neuroinflammation and promote neuronal injury, and pharmacological inhibition of microglia can reduce LPS-induced neurological dysfunction and brain injury [39,45]. In the present study, we examined these cells for a relatively short time, whereas anesthetics are administered over longer periods in clinical settings. Therefore, in such situations, the anesthetic inhibition of glial cell IL-1*β* secretion may provide beneficial results such as cognitive function improvement.

In conclusion, we found that anesthetics, including isoflurane, pentobarbital, midazolam, ketamine, and propofol, suppressed LPS-induced IL-1*β* expression in glial cells and the BV-2 microglial cell line. However, IL-6 and TNF-*α* mRNA expression was not suppressed. Anesthetics commonly suppressed LPS-induced ERK 1/2 phosphorylation, but did not affect NFκB or AP-1 activation. In the mouse experiments, where LPS was injected intraperitoneally, isoflurane suppressed brain IL-1*β* levels and plasma ACTH levels, although IL-1*β* plasma concentration was not changed. Our results indicate that general anesthetics inhibit LPS-induced IL-1*β* upregulation in glial cells, particularly in microglia, and affects HPA axis participation in the stress response.

## Methods

### Animals

This study (ID: Med Kyo 09504) was approved by the Animal Research Committee of Kyoto University (Kyoto, Japan), and all experiments were conducted in accordance with the institutional and NIH guidelines for the care and use of laboratory animals. All procedures were performed on C57BL/6N mice purchased from Japan SLC Inc., Shizuoka, Japan. Food and water were provided *ad libitum*, and the mice were maintained under controlled environmental conditions (24°C, 12-h light/dark cycles).

### Drugs and chemicals

Isoflurane and pentobarbital were obtained from Dainippon Sumitomo Pharma Co., Ltd., Osaka, Japan. 2,6-Diisopropylphenol (propofol), midazolam, PMA, LPS, *E. coli* 055:B5, and PD98059 were obtained from Sigma, St. Louis, MO, USA. Ketamine was purchased from Sankyo Co., Ltd., Tokyo, Japan.

### Cell culture

Primary cultures of cerebral glial cells were prepared from 1- or 2-day-old C57BL/6N CrSlc mice according to a previously described method [46]. Brains were removed from mice under sterile conditions, and the meninges were carefully removed. The tissue was dissociated by passing it through a 320-µm nylon mesh using a rubber policeman. After washing with Hanks’ balanced salt solution that contained DNaseI, the cells were suspended and passed through a 100-µm nylon mesh. Next, the cells were plated on a plastic culture flask (density of two brains per flask) in a 10-ml tissue culture medium. The tissue culture medium contained Dulbecco’s modified Eagle’s medium (DMEM; Gibco/BRL, Gaithersburg, MD, USA) supplemented with 10% fetal bovine serum (FBS), 100 U/ml penicillin, and 0.1 mg/ml streptomycin. The cultures were maintained in a humidified atmosphere of 5% CO_2_ in air at 37°C. The medium was changed after 3 days and then twice weekly thereafter. At the first medium change, the flasks were vigorously shaken to remove oligodendrocytes and their precursors. All *in vitro* experiments were performed with 14-day-old cells. The immortalized mouse microglial cell line BV-2 were cultured in DMEM containing 10% FBS and antibiotics (100 IU/ml penicillin and 100 µg/ml streptomycin) at a density not exceeding 5 × 10^5^ cells/ml, and maintained at 37°C in a humidified incubator with 5% CO_2_ in air. The BV-2 cell is commonly used cell line for the experiment of microglia, but not commercially available. The cell was originally developed by Dr. V. Bocchini (University of Perugia, Perugia, Italy), and it was generously provided to us by Dr. Inoue (Kyusyu University, Fukuoka, Japan). The immortalized murine BV-2 cell line is supposed to exhibit both the phenotypic and functional properties of the reactive microglia cells [47]. 

The A1 astrocyte cell line was purchased from Japanese Collection of Research Bioresources and cultured in DMEM supplemented with 10% FCS, 100 U/ml penicillin, and 0.1 mg/ml streptomycin, and maintained at 37°C in a humidified incubator with 5% CO_2_ in air. THP-1 human myeloid leukemia cells (a gift from Dr. Kume at Kyoto University) were maintained in a RPMI 1640 supplemented with 10% FBS, 100 U/ml penicillin, and 100 g/ml streptomycin at 37°C in a humidified incubator with 5% CO_2_ in air. To promote differentiation to macrophages, THP-1 cells were exposed to PMA at a final concentration of 50 nM for 12 h. The cells then were seeded in six-well plates before being subjected to treatments. In our pilot studies using trypan blue exclusion dye assay, we observed no significant cell death at several time points or with varying doses of LPS and anrsthetics.

### Isoflurane exposure

Isoflurane exposure was performed as previously described [15]. In brief, cell dishes were maintained in airtight chambers housed within a water jacket incubator maintained at 37°C. An in-line calibrated anesthetic agent vaporizer was used to deliver isoflurane to the gas phase of the culture wells. Air was administered at a flow rate of 3 l/min, until the appropriate effluent anesthetic concentration was achieved. Effluent isoflurane concentration was continuously monitored through a sampling port connected to an anesthetic agent analyzer (Capnomac Ultima; Datex-Ohmeda, Helsinki, Finland).

### Reverse transcription and real-time quantitative polymerase chain reaction

RNA was isolated from cells or brains using the FastPure™ RNA Kit (Takara Bio, Inc., Shiga, Japan). First-strand synthesis and real-time RT-PCR were performed using the One Step SYBR™ PrimeScript™ RT-PCR Kit II (Takara Bio) according to the manufacturer’s instructions. PCR was performed using the Applied Biosystems 7300 Real-Time PCR System (Applied Biosystems, Foster City, CA). All PCR primers, except 18S for mouse and IL-1*β* and 18S for human, were purchased from Invitrogen Corporation, Carlsbad, CA. Primer sequences were as follows: IL-1*β*, 5′- CGACAAAATACCTGTGGCCT-3′ (forward) and 5′- TTCTTTGGGTATTGCTTGGG-3′ (reverse); IL-6, 5′-GAAACCGCTATGAAGTTCCTCTCTG-3′ (forward) and 5′- TGTTGGGAGTGGTATCCTCTGTGA-3′ (reverse); TNF-*α*, 5′-GAAAAGCAAGCAGCCAACCA-3′ (forward) and 5′-CGGATCATGCTTTCTGTGCTC -3′ (reverse) 18S for mouse, IL-1*β* and 18S for human primers were purchased from Qiagen (Valencia, CA) (Catalog numbers: 18S mouse: QT02448075; IL-1*β*: QT00001568; 18S human: QT00199367). For each target mRNA, the fold changes in expression were calculated relative to 18S rRNA.

### ELISA of IL-1*β*

Concentrations of IL-1*β* supernatants in glial cell cultures, brains, and plasma of mice were determined using an ELISA kit (R&D Systems Europe, Abingdon, UK). Samples were prepared according to the manufacturer’s instructions. Some modification was made with the brain analysis. In brief, the entire brain was homogenized in phosphate-buffered saline (PBS), centrifuged for 10 min at 5,000 ×*g* at 4°C, and immediately frozen at −20°C. The brain homogenates were assayed after two freeze–thaw cycles to break the cell membranes.

### Immunoblot assay

Whole cell lysates were prepared using ice-cold lysis buffer [0.1% SDS, 1% Nonidet P-40 (NP-40), 5 mM EDTA, 150 mM NaCl, 50 mM Tris-Cl (pH 8.0), 2 mM DTT, 1 mM sodium orthovanadate, and Complete protease inhibitor (Roche Diagnostic, Tokyo, Japan)] following a protocol described previously [15]. The aliquots (100 µg protein) were fractionated by sodium dodecyl sulfate polyacrylamide gel electrophoresis (SDS/PAGE) (7.5% gel) and subjected to an immunoblot assay following a protocol described previously [48]. Primary antibodies raised against IL-1*β* (#8689; Cell Signaling, Stockholm, Sweden), GFAP (#3670; Cell Signaling), IBA-1 (ab107159; abcam, Cambridge, MA), *β*-actin (A5316; Sigma-Aldrich, St. Louis), ERK 1/2 (#4696; Cell Signaling), phospho-ERK1/2 (#9106; Cell Signaling), JNK (#9252; Cell Signaling), phospho-JNK (#9255; Cell Signaling), p38 MAPK (#9212; Cell Signaling), and phospho-p38 MAPK (#9211; Cell Signaling) were used at a 1:1000 dilution. Horseradish peroxidase (HRP)-conjugated sheep anti-mouse immunoglobulin G (IgG) (GE Healthcare, Piscataway, NJ) or donkey anti-rabbit IgG antibodies (GE Healthcare) also were used at a 1:1000 dilution. The signal was detected with enhanced chemiluminescence reagent (GE Healthcare).

### Nuclear protein preparation and Trans-AM assay

Nuclear extracts were prepared from glial cells using a nuclear extraction kit (Active Motif, Carlsbad, CA). Activation of NF-κB and AP-1 was quantified using an ELISA-based assay kit (Trans-AM; Active Motif). The assay was performed following the manufacturer's instructions. Nuclear protein (25 µg) was incubated in a 96-well plate coated with oligonucleotides containing NF-κB consensus site (5′-GGGACTTTCC-3′) or a TPA-response element (TRE) (5′-TGAGTCA-3′). NF-κB and AP-1 contained in nuclear extracts bind specifically to this oligonucleotide during incubation for 2 h at room temperature. The NF-κB p65 antibody or c-jun antibody (100 µl, at 1:1000 dilution) was then added to each well for 1.5 h followed by 100 µl of HRP-conjugated antibody (1:1000 dilution) for 1 h. After adding 100 µl of developing solution for up to 15 min and colorimetric reaction was stopped, the NF-κB and AP-1 activity was determined by reading the absorbance on a spectrophotometer at 450 nm with a reference wavelength of 655 nm.

### Serum ACTH ELISA

Mice were injected intraperitoneally with LPS (5 mg/kg) or saline between 0830 and 1100 h, and were sacrificed 4 h later. Serum ACTH levels were measured using an ACTH kit per the manufacturer’s instructions (MD bioproducts, MN). The minimal detectable ACTH concentration was 0.22 pg/ml.

### Statistical analysis

All data except plasma ACTH concentration are presented as the mean ± SD. Plasma ACTH concentration is presented as the mean ± SEM. Statistical significance was assessed by a Mann–Whitney U-test for between group comparisons, and by a Kruskal–Wallis H-test, followed by a Mann–Whitney U-test with Bonferroni Correction for multiple comparisons. Significance was defined by a P value of <0.05.

## References

[1] Thompson WL, Karpus WJ, Van Eldik LJ (2008) MCP-1-deficient mice show reduced neuroinflammatory responses and increased peripheral inflammatory responses to peripheral endotoxin insult. J Neuroinflammation 5: 35. doi:10.1186/1742-2094-5-35. PubMed: 18706086. 18706086 PMC2527558

[2] McCusker RH, Kelley KW (2013) Immune-neural connections: how the immune system's response to infectious agents influences behavior. J Exp Biol 216: 84-98. doi:10.1242/jeb.073411. PubMed: 23225871. 23225871 PMC3515033

[3] Konsman JP, Parnet P, Dantzer R (2002) Cytokine-induced sickness behaviour: mechanisms and implications. Trends Neurosci 25: 154-159. doi:10.1016/S0166-2236(00)02088-9. PubMed: 11852148. 11852148

[4] Ek M, Kurosawa M, Lundeberg T, Ericsson A (1998) Activation of vagal afferents after intravenous injection of interleukin-1beta: role of endogenous prostaglandins. J Neurosci 18: 9471-9479. PubMed: 9801384. 9801384 10.1523/JNEUROSCI.18-22-09471.1998 PMC6792875

[5] Dantzer R (2004) Cytokine-induced sickness behaviour: a neuroimmune response to activation of innate immunity. Eur J Pharmacol 500: 399-411. doi:10.1016/j.ejphar.2004.07.040. PubMed: 15464048. 15464048

[6] Anforth HR, Bluthe RM, Bristow A, Hopkins S, Lenczowski MJ et al. (1998) Biological activity and brain actions of recombinant rat interleukin-1alpha and interleukin-1beta. Eur Cytokine Netw 9: 279-288. PubMed: 9831177. 9831177

[7] Lenczowski MJ, Bluthé RM, Roth J, Rees GS, Rushforth DA et al. (1999) Central administration of rat IL-6 induces HPA activation and fever but not sickness behavior in rats. Am J Physiol 276: R652-R658. PubMed: 10070124. 10070124 10.1152/ajpregu.1999.276.3.R652

[8] Olson JK, Miller SD (2004) Microglia initiate central nervous system innate and adaptive immune responses through multiple TLRs. J Immunol 173: 3916-3924. PubMed: 15356140. 15356140 10.4049/jimmunol.173.6.3916

[9] Chang Y, Lee JJ, Hsieh CY, Hsiao G, Chou DS, et al. (2009) Inhibitory effects of ketamine on lipopolysaccharide-induced microglial activation. Mediators Inflamm 2009: 705379. 19343193 10.1155/2009/705379 PMC2662525

[10] Nakajima K, Kohsaka S (1998) Functional roles of microglia in the central nervous system. Hum Cell 11: 141-155. PubMed: 10086276. 10086276

[11] Rivest S (2009) Regulation of innate immune responses in the brain. Nat Rev Immunol 9: 429-439. doi:10.1038/nri2565. PubMed: 19461673. 19461673

[12] Gorina R, Font-Nieves M, Márquez-Kisinousky L, Santalucia T, Planas AM (2011) Astrocyte TLR4 activation induces a proinflammatory environment through the interplay between MyD88-dependent NFkappaB signaling, MAPK, and Jak1/Stat1 pathways. Glia 59: 242-255. doi:10.1002/glia.21094. PubMed: 21125645. 21125645

[13] Amor S, Puentes F, Baker D, van der Valk P (2010) Inflammation in neurodegenerative diseases. Immunology 129: 154-169. doi:10.1111/j.1365-2567.2009.03225.x. PubMed: 20561356. 20561356 PMC2814458

[14] Peng M, Wang YL, Wang CY, Chen C (2013) Dexmedetomidine attenuates lipopolysaccharide-induced proinflammatory response in primary microglia. J Surg Res 179: e219-e225. doi:10.1016/j.jss.2012.10.397. PubMed: 22683080. 22683080

[15] Tanaka T, Kai S, Koyama T, Daijo H, Adachi T et al. (2011) General anesthetics inhibit erythropoietin induction under hypoxic conditions in the mouse brain. PLOS ONE 6: e29378. doi:10.1371/journal.pone.0029378. PubMed: 22216265. 22216265 PMC3246473

[16] Ajmone-Cat MA, De Simone R, Nicolini A, Minghetti L (2003) Effects of phosphatidylserine on p38 mitogen activated protein kinase, cyclic AMP responding element binding protein and nuclear factor-kappaB activation in resting and activated microglial cells. J Neurochem 84: 413-416. doi:10.1046/j.1471-4159.2003.01562.x. PubMed: 12559004. 12559004

[17] Lu X, Ma L, Ruan L, Kong Y, Mou H et al. (2010) Resveratrol differentially modulates inflammatory responses of microglia and astrocytes. J Neuroinflammation 7: 46. doi:10.1186/1742-2094-7-46. PubMed: 20712904. 20712904 PMC2936301

[18] Saura J (2007) Microglial cells in astroglial cultures: a cautionary note. J Neuroinflammation 4: 26. doi:10.1186/1742-2094-4-26. PubMed: 17937799. 17937799 PMC2140055

[19] Garwood CJ, Pooler AM, Atherton J, Hanger DP, Noble W (2011) Astrocytes are important mediators of Abeta-induced neurotoxicity and tau phosphorylation in primary culture. Cell Death DIS 2: e167. 21633390 10.1038/cddis.2011.50 PMC3168992

[20] Dinarello CA (2009) Immunological and inflammatory functions of the interleukin-1 family. Annu Rev Immunol 27: 519-550. doi:10.1146/annurev.immunol.021908.132612. PubMed: 19302047. 19302047

[21] Gądek-Michalska A, Bugajski J (2010) Interleukin-1 (IL-1) in stress-induced activation of limbic-hypothalamic-pituitary adrenal axis. Pharmacol Rep 62: 969-982. PubMed: 21273654. 21273654 10.1016/s1734-1140(10)70359-5

[22] Murray CL, Skelly DT, Cunningham C (2011) Exacerbation of CNS inflammation and neurodegeneration by systemic LPS treatment is independent of circulating IL-1beta and IL-6. J Neuroinflammation 8: 50. doi:10.1186/1742-2094-8-50. PubMed: 21586125. 21586125 PMC3119173

[23] Shibakawa YS, Sasaki Y, Goshima Y, Echigo N, Kamiya Y et al. (2005) Effects of ketamine and propofol on inflammatory responses of primary glial cell cultures stimulated with lipopolysaccharide. Br J Anaesth 95: 803-810. doi:10.1093/bja/aei256. PubMed: 16227338. 16227338

[24] Rock RB, Gekker G, Hu S, Sheng WS, Cheeran M, et al. (2004) Role of microglia in central nervous system infections. Clin Microbiol Rev 17: 942-964, table of contents 15489356 10.1128/CMR.17.4.942-964.2004 PMC523558

[25] Wu X, Lu Y, Dong Y, Zhang G, Zhang Y et al. (2012) The inhalation anesthetic isoflurane increases levels of proinflammatory TNF-alpha, IL-6, and IL-1beta. Neurobiol Aging 33: 1364-1378. doi:10.1016/j.neurobiolaging.2010.11.002. PubMed: 21190757. 21190757 PMC3117127

[26] Kress HG (1997) Mechanisms of action of ketamine. Anaesthesist 46(Suppl 1): S8-19. doi:10.1007/PL00002469. PubMed: 9163283. 9163283

[27] Sato Y, Kobayashi E, Murayama T, Mishina M, Seo N (2005) Effect of N-methyl-D-aspartate receptor epsilon1 subunit gene disruption of the action of general anesthetic drugs in mice. Anesthesiology 102: 557-561. doi:10.1097/00000542-200503000-00013. PubMed: 15731593. 15731593

[28] Franks NP, Lieb WR (1994) Molecular and cellular mechanisms of general anaesthesia. Nature 367: 607-614. doi:10.1038/367607a0. PubMed: 7509043. 7509043

[29] Davies PA, Hanna MC, Hales TG, Kirkness EF (1997) Insensitivity to anaesthetic agents conferred by a class of GABA(A) receptor subunit. Nature 385: 820-823. doi:10.1038/385820a0. PubMed: 9039914. 9039914

[30] Matsuda T, Omori K, Vuong T, Pascual M, Valiente L et al. (2005) Inhibition of p38 pathway suppresses human islet production of pro-inflammatory cytokines and improves islet graft function. Am J Transplant 5: 484-493. doi:10.1046/j.1600-6143.2004.00716.x. PubMed: 15707402. 15707402

[31] Dai JN, Zong Y, Zhong LM, Li YM, Zhang W et al. (2011) Gastrodin inhibits expression of inducible NO synthase, cyclooxygenase-2 and proinflammatory cytokines in cultured LPS-stimulated microglia via MAPK pathways. PLOS ONE 6: e21891. doi:10.1371/journal.pone.0021891. PubMed: 21765922. 21765922 PMC3134470

[32] Spooren A, Kooijman R, Lintermans B, Van Craenenbroeck K, Vermeulen L et al. (2010) Cooperation of NFkappaB and CREB to induce synergistic IL-6 expression in astrocytes. Cell Signal 22: 871-881. doi:10.1016/j.cellsig.2010.01.018. PubMed: 20100571. 20100571

[33] Lee HT, Kim M, Kim N, Billings FT, D'Agati VD et al. (2007) Isoflurane protects against renal ischemia and reperfusion injury and modulates leukocyte infiltration in mice. Am J Physiol Renal Physiol 293: F713-F722. doi:10.1152/ajprenal.00161.2007. PubMed: 17596528. Available online at: 10.1152/ajprenal.00161.2007 Available online at: PubMed: 17596528 17596528

[34] Franchi L, Muñoz-Planillo R, Núñez G (2012) Sensing and reacting to microbes through the inflammasomes. Nat Immunol 13: 325-332. doi:10.1038/ni.2231. PubMed: 22430785. 22430785 PMC3449002

[35] Hanamsagar R, Hanke ML, Kielian T (2012) Toll-like receptor (TLR) and inflammasome actions in the central nervous system. Trends Immunol 33: 333-342. doi:10.1016/j.it.2012.03.001. PubMed: 22521509. 22521509 PMC3383346

[36] Hanamsagar R, Torres V, Kielian T (2011) Inflammasome activation and IL-1beta/IL-18 processing are influenced by distinct pathways in microglia. J Neurochem 119: 736-748. doi:10.1111/j.1471-4159.2011.07481.x. PubMed: 21913925. 21913925 PMC3202981

[37] Franchi L, Muñoz-Planillo R, Reimer T, Eigenbrod T, Núñez G (2010) Inflammasomes as microbial sensors. Eur J Immunol 40: 611-615. doi:10.1002/eji.200940180. PubMed: 20201013. 20201013

[38] Park HY, Kim ND, Kim GY, Hwang HJ, Kim BW et al. (2012) Inhibitory effects of diallyl disulfide on the production of inflammatory mediators and cytokines in lipopolysaccharide-activated BV2 microglia. Toxicol Appl Pharmacol 262: 177-184. doi:10.1016/j.taap.2012.04.034. PubMed: 22564536. 22564536

[39] James ML, Wang H, Cantillana V, Lei B, Kernagis DN et al. (2012) TT-301 inhibits microglial activation and improves outcome after central nervous system injury in adult mice. Anesthesiology 116: 1299-1311. doi:10.1097/ALN.0b013e318253a02a. PubMed: 22487803. 22487803

[40] Franchimont D (2004) Overview of the actions of glucocorticoids on the immune response: a good model to characterize new pathways of immunosuppression for new treatment strategies. Ann N Y Acad Sci 1024: 124-137. doi:10.1196/annals.1321.009. PubMed: 15265777. 15265777

[41] Chakravarty S, Herkenham M (2005) Toll-like receptor 4 on nonhematopoietic cells sustains CNS inflammation during endotoxemia, independent of systemic cytokines. J Neurosci 25: 1788-1796. doi:10.1523/JNEUROSCI.4268-04.2005. PubMed: 15716415. 15716415 PMC6725921

[42] Beishuizen A, Thijs LG (2003) Endotoxin and the hypothalamo-pituitary-adrenal (HPA) axis. J Endotoxin Res 9: 3-24. doi:10.1179/096805103125001298. 12691614

[43] Glezer I, Rivest S (2004) Glucocorticoids: protectors of the brain during innate immune responses. Neuroscientist 10: 538-552. doi:10.1177/1073858404263494. PubMed: 15534039. 15534039

[44] Strøm T, Martinussen T, Toft P (2010) A protocol of no sedation for critically ill patients receiving mechanical ventilation: a randomised trial. Lancet 375: 475-480. doi:10.1016/S0140-6736(09)62072-9. PubMed: 20116842. 20116842

[45] Henry CJ, Huang Y, Wynne A, Hanke M, Himler J et al. (2008) Minocycline attenuates lipopolysaccharide (LPS)-induced neuroinflammation, sickness behavior, and anhedonia. J Neuroinflammation 5: 15. doi:10.1186/1742-2094-5-15. PubMed: 18477398. 18477398 PMC2412862

[46] Asou H, Hirano S, Kohsaka S (1989) Changes in ganglioside composition and morphological features during the development of cultured astrocytes from rat brain. Neurosci Res 6: 369-375. doi:10.1016/0168-0102(89)90030-8. PubMed: 2725993. 2725993

[47] Bocchini V, Mazzolla R, Barluzzi R, Blasi E, Sick P et al. (1992) An immortalized cell line expresses properties of activated microglial cells. J Neurosci Res 31: 616-621. doi:10.1002/jnr.490310405. PubMed: 1578513. 1578513

[48] Nishi K, Oda T, Takabuchi S, Oda S, Fukuda K et al. (2008) LPS induces hypoxia-inducible factor 1 activation in macrophage-differentiated cells in a reactive oxygen species-dependent manner. Antioxid Redox Signal 10: 983-995. doi:10.1089/ars.2007.1825. PubMed: 18199003. 18199003

